# Highly Efficient Preparation of Cyclic Dinucleotides *via* Engineering of Dinucleotide Cyclases in *Escherichia coli*

**DOI:** 10.3389/fmicb.2019.02111

**Published:** 2019-09-13

**Authors:** Yun Lv, Qichao Sun, Xiaodan Wang, Yi Lu, Yaoyao Li, Huiqing Yuan, Jing Zhu, Deyu Zhu

**Affiliations:** ^1^Department of Biochemistry and Molecular Biology, School of Basic Medical Sciences, Shandong University, Jinan, China; ^2^Key Laboratory of Chemical Biology (Ministry of Education), School of Pharmaceutical Sciences, Shandong University, Jinan, China; ^3^State Key Laboratory of Microbial Technology, Shandong University, Qingdao, China

**Keywords:** cyclic dinucleotides, dinucleotide cyclases, microbial preparation, affinity purification, adjuvant

## Abstract

Cyclic dinucleotides (CDNs) are widely used secondary signaling molecules in bacterial and mammalian cells. The family of CDNs includes c-di-GMP, c-di-AMP and two distinct versions of hybrid cGAMPs. Studies related to these CDNs require large doses that are relatively expensive to generate by current methods. Here we report what to our knowledge is the first feasible microbial-based method to prepare these CDNs including c-di-GMP, 3′3′-cGAMP and 2′3′-cGAMP. The method mainly includes two parts: producing high yield of CDNs by engineering the overexpression of the proper dinucleotide cyclases (DNCs) and other related proteins in *Escherichia coli*, and purifying the bacteria-produced CDNs by a unified and simple process involving a STING affinity column, macroporous adsorption resin and C18 reverse-phase liquid chromatography. After purification, we obtained the diammonium salts of c-di-GMP, 3′3′-cGAMP and 2′3′-cGAMP with weight purity of >99, >96, >99% and in yields of >68, >26, and >82 milligrams per liter of culture, respectively. This technological platform enables the production of CDNs from cheaper material, provides a sustainable source of CDNs for scientific investigation, and can easily be further developed to prepare CDNs on a large scale for industry.

## Introduction

Since the discovery of c-di-GMP in 1987, cyclic dinucleotides (CDNs) have emerged as highly versatile second messengers that control various important biological processes, such as biofilm development, motility, cell cycle, cell shape and pathogenicity in bacteria, and the innate immune response in mammalian cells (Jenal et al., 2017; Krasteva and Sondermann, 2017). The latest additions to the CDN family are two distinct versions of hybrid cGAMP molecules, 3′3′-cGAMP and 2′3′-cGAMP. 3′3′-cGAMP, which is synthesized by the dinucleotide cyclase DncV (Davies et al., 2012), was originally identified as a second messenger in *Vibrio cholerae*. The distinct 2′3′-cGAMP, which is synthesized by the mammalian cGAMP synthase (cGAS) (Gao et al., 2013a; Sun et al., 2013; Wu et al., 2013; Margolis et al., 2017), is key to innate immune signaling in eukaryotes. These CDNs are widely used in various research fields such as biology to define pathways and molecular mechanisms, pharmacologic and clinical research to fully explore their development as adjuvants in prophylactic vaccines against infections, and in therapeutic immunizations against cancer (Krasteva and Sondermann, 2017; Mankan et al., 2017; Shae et al., 2019).

Currently, CDNs are manufactured entirely from chemical or enzymatic synthesis (Romling et al., 2013). The chemical synthesis approaches are time-consuming and environmentally unsustainable because multiple steps and hazardous chemicals are required (Gaffney et al., 2010; Gaffney and Jones, 2012; Gao et al., 2013a; Schwede et al., 2017). In contrast, the enzymatic synthesis of CDNs by using the corresponding dinucleotide cyclases (DNCs) is relatively simple and safe. The first member of the CDN family, c-di-GMP, can now be enzymatically synthesized at gram scale (Rao et al., 2009; Spehr et al., 2011; Venkataramani and Liang, 2017). However, the enzymatic synthesis of c-di-GMP has some drawbacks because the purification of diguanylate cyclases (DGCs) is laborious and the precursor GTP is relatively expensive or formed from economical starting materials by using extra enzymatic steps (Spehr et al., 2011). The studies on enzymatic production of the two latest members of the CDN family, 3′3′-cGAMP and 2′3′-cGAMP, have just begun and also use the expensive precursor GTP (Davies et al., 2012; Diner et al., 2013; Zhang et al., 2013). With the growing interest in studying these CDNs, we were compelled to develop new, more economical and efficient methods to produce these signaling molecules.

Additionally, there is another approach that can prepare CDNs directly from bacteria. However, this approach has been hampered by low intracellular concentrations of CDNs in bacteria and lack of effective purification method. The intracellular concentration of CDNs are regulated by DNCs and phosphodiesterase (PDE) (Kalia et al., 2012; Zheng et al., 2013). For example, in *Escherichia coli* c-di-GMP levels are regulated by four DGCs that synthesize c-di-GMP and one PDE (YhjH) that degrades it Boehm et al. (2010). Whereas c-di-GMP concentrations were below the detection limit in wild type *E. coli* cells (<0.2 pmol mg^–1^ cells), c-di-GMP concentrations varied in mutant strains and correlated with the protein expression levels of the active DGCs and PDEs (Simm et al., 2004). The Jenal lab mutated *C. crescentus* DgcA DGC to abolish product inhibition and showed that only overexpression of this mutant enzyme in *E. coli* strain BL21 resulted in a relatively higher cellular level of c-di-GMP (1.57 nmol mg^–1^ dry weight cells) (Christen et al., 2006). In addition, the Mekalanos group and our group have shown a strong LC-MS signal for 3′3′-cGAMP in *E. coli* expressing wild type DncV (Davies et al., 2012; Zhu et al., 2014). Moreover, microbial production through engineering product-specific enzymes or metabolic pathways has advanced as an attractive alternative to chemical synthesis in recent years with many successful applications reported (Keasling, 2010; Zhang et al., 2012; Chubukov et al., 2016). For these reasons, we sought a feasible microbial-based method to prepare these CDNs.

Here, we have successfully established a highly efficient microbial-based method, which to our knowledge is the first report of preparing CDNs directly from bacteria culture. First, we achieved the high yield microbial production of CDNs including c-di-GMP, 3′3′-cGAMP and 2′3′-cGAMP by engineering the overexpression of the proper DNCs and other related proteins in *E. coli*. The yields of c-di-GMP, 3′3′-cGAMP and 2′3′-cGAMP reached 33.03 and 13.81 nmol mg^–1^ wet weight cells and 217.04 μmol/L culture volume, respectively. Then we developed a unified and simple purification process for separating CDNs directly from bacterial or growth medium. Finally, we obtained the high purity diammonium salts of c-di-GMP, 3′3′-cGAMP and 2′3′-cGAMP in yields of >68, >26, and >82 milligrams per liter of culture. The high yield of crude CDNs and simple purification make the method very economical and practical. In addition, because the CDNs are the universal signaling molecules in bacterial and mammalian cells, our purification strategy can be extended to a platform for the detection of the intracellular concentrations of CDNs in cells.

## Materials and Methods

### Plasmid Construction and Transformation

The gene sequences encoding full-length mouse cGAS (mcGAS) or the carboxy-terminal domain (residues 149–379) of human STING^H232^ (STING^CTD^) were optimized, synthesized (Beijing AuGCT DNA-SYN Biotechnology, Co., Ltd., China) and received as a gift from Dr. Ye Xiang (Tsinghua University). The gene sequences encoding full-length *Staphylococcus aureus* guanylate kinase (GMK), full-length *E. coli* nucleoside diphosphate kinase (NDK), and residues 82–248 of *Thermotoga maritima* DGC with an Arg158Ala mutation (tDGCm) were optimized and synthesized (Shanghai Generay Biotech, Co., Ltd., China). The gene sequence encoding residues 1–419 of vc0179 (DncVt) were amplified from genomic DNA isolated from *V. cholera* O1 biovar E1 Tor Strain N16961.

All expression plasmids were constructed using conventional restriction-ligation cloning procedures or QuikChange site-directed mutagenesis (Braman et al., 1996) (Supplementary Table 1). All gene sequences were confirmed by DNA sequencing (Shanghai Biosune Bio, Co., Ltd., China). The primers used for cloning and mutagenesis are listed in Supplementary Table 2. *E. coli* DH5α, BL21(DE3), and BL21-CondonPlus(DE3)-RIL strains (Agilent Technologies, Stratagene) were used for cloning and expression studies. As for the expression, the target expression plasmids were individually transformed or co-transformed into *E. coli* strain BL21 (DE3) or BL21-CondonPlus(DE3)-RIL. For production of 2′3′-cGAMP, mcGAS expression vectors were transformed into *E. coli* strain BL21-CodonPlus (DE3)-RIL (Supplementary Table 1).

### CDNs Production

All transformed cells were first grown overnight at 37°C in Luria-Bertani (LB) medium containing appropriate antibiotics. When inoculated into fresh LB or TB medium with the corresponding antibiotics, cells from an overnight culture were diluted 100-fold and grown in the fresh medium. When inoculated into fresh unmodified or modified M9 minimal medium (Paliy and Gunasekera, 2007; Walden, 2010), the cells from 1 volume of the overnight culture were pelleted by centrifugation, washed twice, resuspended in 1 volume of M9 minimal medium, and transferred to 250 volumes of fresh unmodified or modified M9 minimal medium. Then, all of the inoculation cultures were incubated at 37°C with shaking. When the cultures reached an OD600 value from 0.6 to 1.2, cells were induced by the addition of 0.1 mM isopropyl β-D-1-thiogalactopyranoside (IPTG) and followed by incubation at varying temperatures (18, 25, or 37°C) for 6, 12, 20, or 36 h. Finally, the bacterial cultures were centrifuged at 4500 rpm for 30 min at 4°C, and the cell pellets or culture supernatants were retained or stored at −80°C for later use.

### HPLC and HPLC-MS/MS Analysis of the CDNs

For the analysis of the initial production of the intracellular CDNs in bacteria, the cell pellets were resuspended in a corresponding volume of water according to the ratio (10 volumes of bacterial culture for harvesting these cells/1.8 volume of water) (Supplementary Table 3), heated at 100°C for 15 min, and centrifuged (16,000 *g* for 15 min) to obtain the sample supernatant. For the analysis of the initial secreted production of the 2′3′-cGAMP, the cell culture medium was cleared by centrifugation (16,000 *g* for 15 min) to obtain the sample supernatant. To characterize the final purified CDNs (in diammonium salt form), a stock solution of each CDN was prepared by dissolving the resultant CDN powders in pure water and further cleared by centrifugation (16,000 *g* for 15 min) to obtain the sample supernatant.

The entire sample supernatant was analyzed by HPLC or HPLC-MS/MS. High resolution LC/MS analysis was performed on a Thermo LTQ velos pro Orbitrap ETD spectrometer equipped with an HPLC system (SHIMADZU, prominence LC-20AB) using an electrospray (ESI) ionization source in positive mode. Ionization source parameters were set to the following: capillary voltage, 3500 kV; sheath gas flow rate, 20 arb; aux gas flow rate, 5 arb; capillary temperature, 275°C. Full scan mass spectra were acquired in mass range from M/Z 300–800 at 60,000 resolution at m/z = 400 in the Orbitrap. A YMC-pack pro-C18 reverse phase column (4.6 mm × 250 mm, 5 μm, YMC, Japan) was used to separate samples. Samples were monitored by UV 254 nm. Phase A contained 5 mM ammonium acetate (pH 5.0) and Phase B contained 100% acetonitrile. The samples were eluted using a linear gradient from 1.5 to 10% B at a flow rate of 1 ml/min over 15 min. To enable a more in-depth examination, tandem MS/MS spectra (resolution 30,000 at m/z = 400) were obtained using higher-energy collision dissociation (HCD). Ions were selected using an iso width (approximately 1 m/z) and normalized collision energy set to 24.

In order to roughly estimate the initial concentration of CDNs produced in bacteria culture, we generated a c-di-GMP HPLC standard curve and obtained the linear regression equation (Supplementary Figure 1). The commercial standard of c-di-GMP was obtained from Biolog Life Science Institute (Bremen, Germany) and used without further purification. 20 mM stock solutions of commercial c-di-GMP were prepared by dissolving the c-di-GMP powder in pure water and diluting them to produce standard concentrations (7.8125, 15.625, 31.25, 62.5, 125, 500, and 1000 μM). Each standard was injected with 2 μL volume per HPLC run (UV 254 nm). A c-di-GMP HPLC standard curve was prepared by plotting the c-di-GMP amount in pmol vs. the corresponding peak areas (unit, uAU × s), which was determined using the software LCsolution 1.26 SP 1 of the HPLC system. Based on the linear regression equation obtained from the c-di-GMP HPLC standard curve and the extinction coefficient at maximum absorbance (^1^ ε for c-di-GMP, 23700 M^–1^ cm^–1^ at 253 nm; ε for two cGAMP, 25050 M^–1^ cm^–1^ at 256 nm), the initial concentration of CDNs produced by bacteria were roughly calculated using corresponding different equations (for details see the legend of Supplementary Table 3).

### Preparation of STING-Immobilized Affinity Resin

The gene of the ligand-binding domain of human STING^R232^(STING^LBD^, residues 149–341) was amplified by standard PCR procedure and subcloned into the pET-30a (Novagen) between *Nde*I and *Xho*I sties, resulting in a C-terminal hexahistidine tag (Supplementary Tables 1, 2). The recombinant plasmid was then transformed into *E. coli* BL21 (DE3) competent cells to obtain the STING^LBD^ expression strain, which was grown overnight at 37°C in LB medium containing 50 μg/ml kanamycin. The overnight culture was used to inoculate fresh LB medium (1:100 dilution) supplemented with 50 μg/ml kananmycin and grown at 37°C to an OD600 of 0.7–0.8 before being induced with 0.1 mM IPTG at 18°C for 16 h. Cells from 16 liters of culture were collected by centrifugation, resuspended in 650 ml of resuspension buffer (20 mM Tris-HCl pH8.0, 200 mM NaCl) supplemented with 0.2 mg/ml DNase I and 0.1 mM Phenylmethylsulfonyl fluoride (PMSF), and then lysed using a mini ultra high pressure cell disrupter (JNBIO, China). The cell lysate was clarified by centrifugation (12,000 *g* for 60 min at 4°C), and the supernatant was applied onto a column with 50 ml Ni-Chelating Sepharose Fast Flow resin (IMAC, cat. no. 17057502, GE Healthcare, United States), which was pre-equilibrated in the resuspension buffer. After loading of the STING^LBD^ protein, the resin was washed with 2 L of the resuspension buffer supplemented with 20 mM imidazole followed by 1 L of the resuspension buffer, 0.5 L of 2M urea and 0.5 L of the resuspension buffer, and was ready for use (we named the resin as STING-immobilized affinity resin).

The test results have demonstrated that the STING-immobilized affinity resin is stable in the resuspension buffer and can be stored for up to 1 month under the temperatures of 4 to 10°C while replacing the resuspension buffer every day to avoid microbial growth. The 50 ml of STING-immobilized affinity resin can specifically bind up to ∼30 mg of CDN, which can be eluted by urea solution. After use, the resin should be washed with 0.5 L of 2M urea and 0.5 L of the resuspension buffer to be ready for reuse. The reuse of the STING-immobilized affinity resin can also be performed with different CDNs. Based on our experience, the CDN binding capacity of the resin was obviously reduced after reusing more than 15 times, so we recommend a maximum of 15 runs per column. This follows a common regeneration procedure whereby Ni-chelating resin removes the STING protein.

### Preparation of CDNs

#### Production of CDNs

The production of CDNs is made by using the same protocol as described in the above paragraph (**CDNs Production**). The details of production of each CDN are presented in Section “Results and Discussion” as well as in the Supplementary Methods.

#### Purification of CDNs

In brief, the harvested cell pellets harboring c-di-GMP or 3′3′-cGAMP or the culture supernatant harboring 2′3′-cGAMP were pretreated to obtain the sample supernatant containing target CDN, which was purified by sequential chromatography using STING-immobilized affinity resin, macroporous adsorption resin SP207 and C18 ODS-AQ semi-preparative column. Then, the purified CDNs were neutralized, dried and characterized. The details of the purification of CDNs are also presented in Results and Discussion and in the Supplementary Methods.

### Determination of the Weight Purity of the Purified CDNs

The weight purity of the purified CDNs was measured by Unico UV-2000 spectrophotometry (Unico, United States) using the respective extinction coefficient at maximum absorbance. A stock solution of 10 mM CDNs (diammonium salt) was prepared by dissolving the corresponding resultant CDN powder (diammonium salt) in pure water and cleared by centrifugation (16,000 *g* for 15 min). All stock solutions were diluted 500 times with water to produce 20 μM working solution for the UV absorption measurements. Finally, the relative weight purity of the CDN samples was calculated using the following equation:

(1) $$P\left(\%\right)=\left(A\times 10^{8}\right)/\left[\varepsilon \times L\left(cm\right)\times 20\left(\mu M\right)\right]$$

where A, ε, L, and 20 μM represent the measured absorption value of the 20 μM working solutions of the purified CDNs (diammonium salt) at the corresponding maximum wavelength (c-di-GMP at 253 nm; two cGAMPs at 256 nm), molar extinction coefficient ε described above, path length of the beam of light through the sample (cuvette length, unit is cm), and the theoretical concentration, respectively.

### Expression and Purification of STING^R232/CTD^

The carboxy-terminal domain of human STING R232 (STING^R232/CTD^) was expressed as described in the above paragraph and reference (Shang et al., 2012). The STING^R232/CTD^ protein was purified using a Ni- Chelating Sepharose Fast Flow resin and eluted with 20 mM Tris-HCl (pH 8.0) buffer containing 200 mM NaCl and 250 mM imidazole. Subsequently, the eluted protein was concentrated and injected onto a gel-filtration column (Superdex 200 10/300 GL, GE Healthcare) with a running buffer containing 25 mM HEPES (pH 7.8) and 150 mM NaCl. The peak fractions of the protein were pooled at a concentration of 4.5 mg/ml, flash-frozen in liquid nitrogen, and stored at −80°C for binding test.

### STING Binding Test by Isothermal Titration Calorimetry (ITC)

Isothermal titration calorimetry (ITC) was employed to measure the binding affinities between STING^R232/CTD^ and the purified CDNs using a MICROCAL PEAQ-ITC (Malvern Instruments, United Kingdom) (Zhang et al., 2013; Du and Su, 2017). The purified STING^R232/CTD^ was thawed and diluted to 100 μM in a buffer containing 25 mM HEPES pH 7.8 and 150 mM NaCl. Next, it was loaded into the sample cell. A 50 mM stock solution of the CDN was prepared in pure water, diluted to 1 mM (0.8 mM for 3′3′-cGAMP) with the same buffer as the STING^R232/CTD^, and loaded into the syringe injector. Titrations were performed at 25°C and involved 19 injections (1 × 0.4 and 18 × 2 μl) at 150 s intervals. A reference titration of ligand into buffer was used to correct for heat of dilution. Data fitting was based on a single-site binding model using the Origin software package (MicroCal). The dissociation constant was derived from the data by using standard procedures.

## Results and Discussion

### Production of c-di-GMP

**FIGURE 1:**
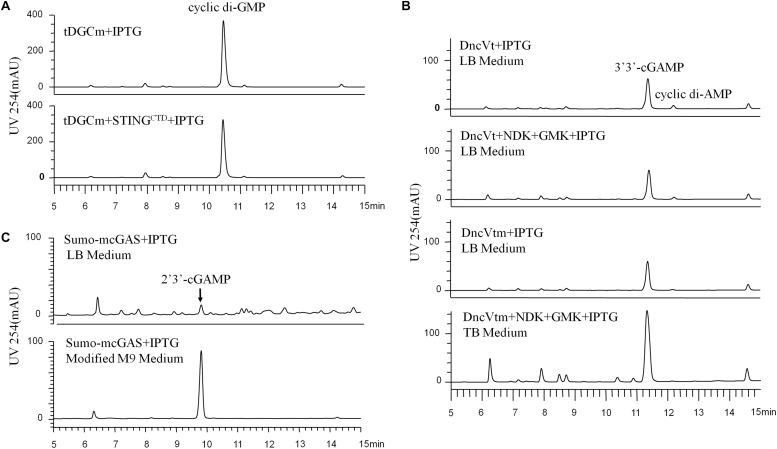
HPLC analysis (UV 254 nm) of the initial CDNs before purification, which were produced by the different engineered *Escherichia coli* cells expressing the proper DNCs and other related proteins under various conditions. **(A)** Production of c-di-GMP in *E. coli* BL21 (DE3) cells expressing only tDGCm or co-expressing tDGCm and STING^CTD^ under the optimized conditions. **(B)** Production of 3′3′-cGAMP in *E. coli* BL21 (DE3) cells: top, cells were grown in LB medium and expressed only the DncVt; second from top, cells were grown in LB medium and co-expressed DncVt, GMK, and NDK; third from top, cells were grown in LB medium and expressed only the DncVtm; bottom, cells were grown in TB medium and co-expressed DncVtm, GMK, and NDK. **(C)** The secreted production of 2′3′-cGAMP in *E. coli* BL21-CodonPlus (DE3)-RIL cells expressing mcGAS as a SUMO fusion protein in LB or modified M9 minimal medium with the other optimized conditions.

**FIGURE 2:**
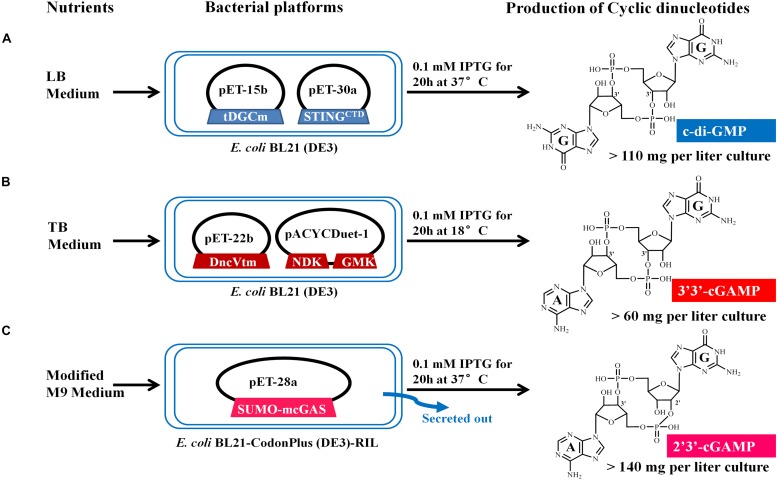
Schematic representation of the microbial production of c-di-GMP **(A)**, 3′3′-cGAMP **(B)**, and 2′3′-cGAMP **(C)** via engineering the expression of the proper DNCs and other related proteins in *E. coli*. The expression vectors and inserted genes are simply indicated. Gene symbols and the enzymes they encode: tDGCm, DGC Arg158Ala mutant from *Thermotoga maritime*; STING^CTD^, carboxy-terminal domain of human STING^H232^; DncVtm, mutant Thr179Arg of *V. cholera* DncV truncation; NDK, nucleoside-diphosphate kinase from *E. coli*; GMK, guanylate kinase from *Staphylococcus aureus*; SUMO-mcGAS, mouse cGAS as a SUMO fusion protein. Three CDNs structural drawings depict the free acid form of the phosphate moieties.

We chose *E. coli* BL21 (DE3) as the host strain, which is used universally with ease of manipulation, has no detectable CDNs in the wild type cells, and was demonstrated previously to be appropriate for high expression of bacterial DGCs (Simm et al., 2004; Christen et al., 2006; Zhu et al., 2014). Previous studies have shown that *Thermotoga maritima* DGC R158A mutant (tDGCm) weakens product inhibition considerably and may be the most efficient enzyme currently available for the large-scale enzymatic production of c-di-GMP (Rao et al., 2009; Venkataramani and Liang, 2017). Therefore, we synthesized and expressed a codon-optimized version of tDGCm (residues 82–248) in *E. coli* BL21 (DE3) (Supplementary Table 1). As expected, under the optimized induction condition for c-di-GMP production (shaking for 20 h at 37°C with 0.1 mM IPTG in LB medium), the expression of the synthetic *tDGCm* gene in *E coli* BL21 (DE3) resulted in an intracellular concentration of c-di-GMP of up to 35.64 nmol mg^–1^ wet weight cells [initial yield, 133.66 mg (in free acid) per liter of culture, Figure 1A and Supplementary Table 3], but the yield of the following purification procedure has a low reproducibility as the process of treating cells may alter the c-di-GMP levels (Roy et al., 2013). We also examined whether the c-di-GMP intracellular concentration could be increased by co-expressing tDGCm with a c-di-GMP receptor protein such as PilZ domain (residues 1–122) from *Pseudomonas aeruginosa* alginate biosynthesis protein Alg44 (Whitney et al., 2015) or carboxy-terminal domain of human STING^H232^ (STING^CTD^, residues 149–379, Supplementary Table 1) (Burdette et al., 2011; Huang et al., 2012; Ouyang et al., 2012; Shang et al., 2012; Shu et al., 2012; Yin et al., 2012). Results showed that the c-di-GMP intracellular concentration was obviously decreased by co-expression with PilZ domain and slightly reduced by co-expression with STING^CTD^ (33.03 nmol mg^–1^ wet weight cells, 114.36 mg (in free acid) per liter of culture, Figure 1A and Supplementary Table 3). Interestingly, the cells co-expressing tDGCm and STING^CTD^ had a high reproducibility of the c-di-GMP yield for the following purification procedure. Therefore, the cells co-expressing tDGCm and STING^CTD^ in LB medium with 0.1 mM IPTG at 37°C for 20 h were chosen for the final protocol (Figure 2A).

### Production of 3′3′-cGAMP

We stimulated endogenous 3′3′-cGAMP production by expressing the truncated *V. cholera* DncV (DncVt, residues 1–419) in *E. coli* BL21 (DE3) (Supplementary Table 1). DncVt is more stable than the full length DncV and retains the enzyme activity (Zhu et al., 2014). As expected, when the *E. coli* BL21 (DE3) strain harboring the expression vector of DncVt was cultured in LB medium and induced with 0.1 mM IPTG followed by further incubation at 18°C for 20 h, the 3′3′-cGAMP intracellular concentration in these cells reached up to 6.54 nmol mg^–1^ wet weight cells [21.96 mg (in free acid) per liter of culture, Figure 1B and Supplementary Table 3]. However, consistent with the previous studies (Davies et al., 2012; Zhu et al., 2014), we also observed an obvious c-di-AMP product that accounted for about 9.5% of the desired 3′3′-cGAMP product (Figure 1B), and we found it difficult to completely remove the c-di-AMP by a simple purification method. Because DncV preferentially synthesizes a hybrid cGAMP from ATP and GTP, we hypothesized that reducing ATP levels and increasing GTP levels may reduce the undesired c-di-AMP *in vivo*. Previous *in vitro* studies have shown that guanylate kinase (GMK) catalyzes the ATP-dependent phosphorylation of GMP into GDP, and nucleoside-diphosphate kinase (NDK) catalyzes the reversible exchange of gamma-phosphate between nucleoside triphosphates and diphosphates to produce nucleotide triphosphates. Together, an efficient enzyme cascade for synthesizing GTP from GMP and ATP can be formed (Spehr et al., 2011). We therefore synthesized codon-optimized versions of *Staphylococcus aureus GMK* gene (Liu et al., 2015) and *E. coli NDK* gene (Moynie et al., 2007) and co-expressed them with DncVt in *E. coli* strain BL21 (DE3) (Supplementary Table 1). Compared to cells expressing only DncVt, the intracellular 3′3′-cGAMP concentration did not obviously change in cells co-expressing DncVt, GMK and NDK, but c-di-AMP production was reduced to about 5.8% of 3′3′-cGAMP production (Figure 1B and Supplementary Table 3). To further decrease c-di-AMP production, we examined crystal structures of DncV (Kranzusch et al., 2014; Ming et al., 2014; Zhu et al., 2014; Kato et al., 2015) and designed DncVt mutants to disfavor c-di-AMP production. We found that expressing DncVt mutant Thr179Arg (here named DncVtm) reduced the c-di-AMP production to 2.8% of the 3′3′-cGAMP production (Figure 1B and Supplementary Table 3). In addition, we found that using Terrific Broth (TB) medium increased the cell mass and 3′3′-cGAMP production. Therefore, the cells co-expressing DncVtm, GMK, and NDK in TB medium with 0.1 mM IPTG at 18°C for 20 h were chosen for the final protocol (Figure 2B and Supplementary Table 1). As a result, c-di-AMP production was reduced to about 0.4% of the 3′3′-cGAMP production, and the intracellular concentration of 3′3′-cGAMP increased to 13.81 nmol mg^–1^ wet weight cells [initial yield, 64.23 mg (in free acid) per liter of culture, Figure 1B and Supplementary Table 3].

### Production of 2′3′-cGAMP

2′3′-cGAMP is produced by a cytoplasmic DNA sensor cGAS in mammalian cells and functions as an endogenous metazoan second messenger to stimulate innate immunity. There have been no reports to show the production of 2′3′-cGAMP in bacterial cells. As we know, the recombinant cGAS proteins retain DNA-dependent 2′3′-cGAMP cyclase activity *in vitro* after expression and purification from *E. coli* (Sun et al., 2013), and some of the *E. coli* cellular DNA are of naked DNA that are not encumbered by specific binding proteins (von Hippel, 2007). Therefore, we hypothesized that the cellular cGAS overexpressing in *E. coli* could bind to and be activated by the naked DNA and produce the 2′3′-cGAMP. We chose the *E. coli* BL21-CodonPlus (DE3)-RIL strain as the host strain that was demonstrated previously to be appropriate for high expression of cGAS (Gao et al., 2013a), synthesized a codon-optimized version of full-length mouse *cGAS (mcGAS)* gene, and expressed mcGAS as a SUMO fusion protein in *E. coli* strain BL21-CodonPlus (DE3)-RIL (Supplementary Table 1). Unexpectedly, LC/MS analysis identified 2′3′-cGAMP in the LB culture medium (∼26.39 μM, Figure 1C and Supplementary Figure 2), but not in the harvested cells. The mechanism of secretion of 2′3′-cGAMP by *E. coli* is unclear.

To simplify the purification process of 2′3′-cGAMP, we tested production and growth of cells in a standard M9 minimal medium supplemented with several essential amino acids. To our pleasant surprise, HPLC analysis of the culture medium showed that the amount of 2′3′-cGAMP in M9 minimal medium was greater than that in LB medium, and as expected, the level of impurity in M9 minimal medium was obviously lower. To further improve the production of 2′3′-cGAMP, we slightly modified the contents of the M9 minimal medium. The optimal modified M9 minimal medium was M9 minimal salts supplemented with 0.8% glucose, 5 mM MgSO_4_, 0.1 mM CaCl_2_, and 0.01 mM ferrous sulfate that must be made fresh and is a key factor for high production of 2′3′-cGAMP. The optimal induction temperature and time was 37°C for ≥20 h. Therefore, *E. coli* strain BL21-CodonPlus (DE3)-RIL expressing Sumo-mcGAS in the optimal modified M9 minimal medium with 0.1 mM IPTG at 37°C for 20 h was chosen for the final protocol (Figure 2C and Supplementary Table 1). Under these selected conditions, the 2′3′-cGAMP concentration in the modified M9 minimal medium reached up to 217.04 μM [initial yield, 146.28 mg (in free acid) per liter of culture], which was about 8.22 times the concentration in LB medium (Figure 1C and Supplementary Table 3).

### Purification of CDNs

**FIGURE 3:**
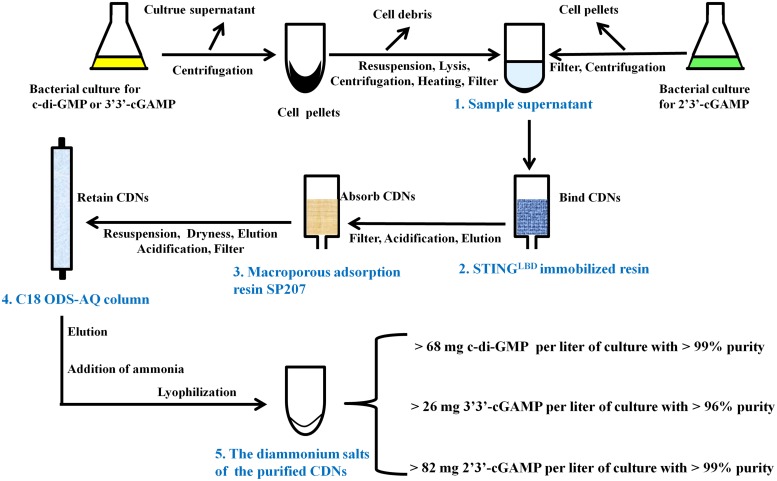
Schematic representation of CDNs purification from the bacteria culture.

There are many literature reports on purification of CDNs from enzymatic or chemical reaction mixture (Gaffney et al., 2010; Spehr et al., 2011; Gaffney and Jones, 2012; Zheng et al., 2013; Venkataramani and Liang, 2017) and some on quantification of CDNs from bacterial or mammal cells (Roy et al., 2013; Almine et al., 2017), but to date no feasible purification method of CDNs from bacteria has been developed. To purify CDNs from the harvested cells or growth medium, we developed a unified, simple and feasible purification method by a great deal of experiments, which are outlined in Figure 3 and briefly described in the following paragraph (for further details see Supplementary Methods).

#### Preparation of the Sample Supernatant Containing CDNs From the Bacterial Culture

As for the c-di-GMP or 3′3′-cGAMP, the bacterial culture was centrifuged, the supernatant was discarded, the harvested cell pellets were thoroughly resuspended in the resuspension buffer (20 mM Tris-HCl pH8.0 and 200 mM NaCl) supplemented with 0.2 mg/ml DNase and 0.1 mM PMSF and lysed by using a low temperature ultra-high pressure cell disrupter (JNBIO, China). Cell debris was removed by centrifugation, the supernatant was retained, heated at 100°C, centrifuged again, and filtered through a PES sterile syringe filter (pore size 0.22 μm, Millipore, United States) to produce the sample supernatant containing c-di-GMP or 3′3′-cGAMP. As for 2′3′-cGAMP, the bacterial culture was centrifuged and the culture supernatant was retained and filtered through cellulose filters (pore size 0.45 μm) to produce the sample supernatant containing 2′3′-cGAMP.

#### Preliminary Purification of CDNs by STING-Immobilized Affinity Resin

The ligand-binding domain of human STING^R232^ (STING^LBD^, residues 149–341) (Shang et al., 2012; Gao et al., 2013b) with a C-terminal His-tag was bound to Ni-chelating Sepharose Fast Flow resin (GE Healthcare) to yield a reusable STING^LBD^ immobilized affinity resin as described in Section “Materials and Methods.” Each sample supernatant containing CDNs was loaded onto and captured by the STING^LBD^ immobilized affinity resin, which was pre-equilibrated in the resuspension buffer. After extensive washing with 300 mM sodium acetate, each target CDN was eluted from the resin by urea solution (0.1M urea for c-di-GMP or 3′3′-cGAMP and 1.5M urea for 2′3′-cGAMP).

#### Solid Phase Extraction of CDNs by Macroporous Adsorption Resin SP207

The elution from the STING^LBD^ immobilized affinity resin was acidified to about pH3.0 with acetic acid, filtered through cellulose filters (pore size 0.45 μm), and incubated with a pre-acidified macroporous adsorption resin SP207 (Diaion SP207, Mitsubishi Chemical Industries, Ltd., Japan). Acidification is important for improving the selective CDNs’ adsorption capacity for macroporous resin. After incubating for 1 h, the SP207 resin was transferred to a column and washed with 1% (v/v) acetic acid and H_2_O. The target CDNs were eluted by 10% ethanol solution.

#### Further Purification of CDNs by C18 ODS-AQ Column

The elution from the macroporous adsorption resin was dried by rotary evaporation, resuspended in H_2_O (pH 9, adjusted with ammonium hydroxide), filtered through a PES sterile syringe filter (pore size 0.22 μm), acidified to pH 2.1 with HCl (pH 4.5 for 2′3′-cGAMP), and loaded onto a YMC-Pack C18 ODS-AQ semi-preparative column (10 mm × 250 mm, 5 μm, YMC, Japan) pre-equilibrated in AQ-A buffer (5 mM ammonium formate adjusted to pH 2.1 with formic acid for c-di-GMP or 3′3′-cGAMP, or pH4.5 for 2′3′-cGAMP). After washing with AQ-A buffer, the CDNs were eluted using a gradient from 0 to 100% B (AQ-B buffer, 10% acetonitrile) (Supplementary Figure 3).

#### Preparation of the Diammonium Salts of the Purified CDNs

The pooled peak fractions corresponding to the target CDNs from YMC-Pack C18 ODS-AQ semi-preparative column were neutralized with ammonium hydroxide, dried by rotary evaporation, resuspended in 0.5 ‰(v/v) ammonium hydroxide solution, and freeze-dried by a vacuum-freezing dryer (Heto-Holten, Denmark). As a result, we obtained the diammonium salts of c-di-GMP, 3′3′-cGAMP and 2′3′-cGAMP in yields of >68, >26, and >82 milligrams per liter of culture, which correspond to overall yields of >56, >38, and >53% of the initial production in bacterial culture, respectively.

**FIGURE 4:**
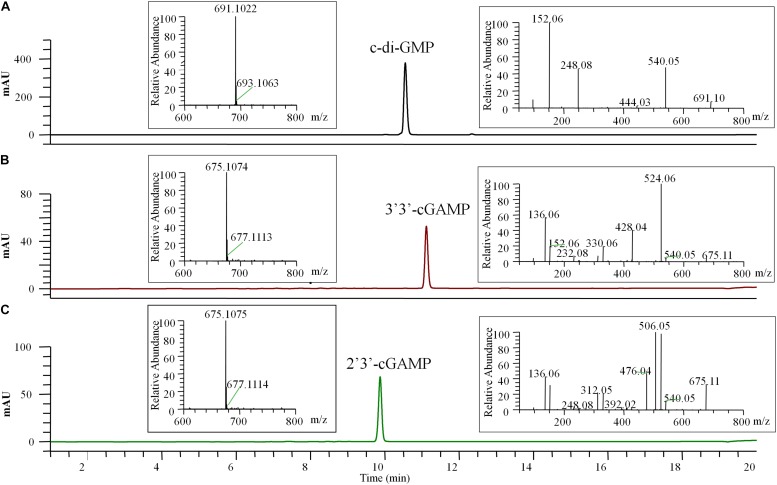
HPLC-MS/MS chromatogram (UV 254 nm) of the final pure c-di-GMP **(A)**, 3′3′-cGAMP **(B)**, and 2′3′-cGAMP **(C)**. The left insert panels show the MS spectra of the final pure CDNs, the ions at m/z 691.1022 (calculated mass 691.1027), 675.1074 (calculated mass 675.1078), and 675.1075 (calculated mass 675.1078) represent the [M + H]^+^ ion of c-di-GMP, 3′3′-cGAMP and 2′3′-cGAMP, respectively. The right insert panels show the tandem mass spectra of the final pure CDNs resulting from higher-energy collision dissociation (HCD) of the precursor ion ([M + H]^+^ = 691.10 or 675.11).

The final prepared diammonium salts of CDNs had the appearance of white powders and were characterized by HPLC-MS/MS and STING binding affinity test. Their weight purity was measured by UV spectroscopy using the estimated extinction coefficient at maximum absorbance (^1^ε for c-di-GMP, 23700 M^–1^ cm^–1^ at 253 nm; ε for two cGAMP, 25050 M^–1^ cm^–1^ at 256 nm) and calculated using the Eq. (1) in the Section “Materials and Methods.” LC/MS clearly identified the pure products as the target CDNs, and the identification of 3′3′-cGAMP and 2′3′-cGAMP was inferred by the requisite enzymes (DncVtm, mcGAS) and supported by the tandem mass (MS/MS) spectra (Figure 4). No obvious impurities were detected by HPLC with UV detection at the 254 nm wavelength, and the relative weight purity of the final resultant c-di-GMP, 3′3′-cGAMP and 2′3′-cGAMP are 99.86 ± 0.52, 96.88 ± 0.38, and 99.53 ± 0.34%, respectively. Consistent with previous reports (Burdette et al., 2011; Shang et al., 2012; Gao et al., 2013b; Zhang et al., 2013), the purified c-di-GMP or 3′3′-cGAMP bound to STING had a dissociation constant K_d_ of 4.09 or 5.40 μM, and the purified 2′3′-cGAMP to STING is endothermic with a dissociation constant K_d_ of ∼16 nM, but at this high affinity, curve fitting may not be precise (Supplementary Figure 4).

## Conclusion

Our results show that the microbial-based preparation method of CDNs is practical and much more economical than chemical or enzymatic synthesis methods. We have made every effort to keep our protocol as efficient as possible, however additional optimization may improve the yields in the future, especially for the yields of 3′3′-cGAMP. Due to the ease of cultivating bacteria and the simplicity of our purification procedure, we believe that the method we described in this paper can be used immediately to prepare sufficient CDNs for scientific investigation at the laboratory scale and can easily be further developed to prepare CDNs on a larger scale to meet the requirements for potential medical applications.

We did not explore a similar method for production of c-di-AMP because it can be synthesized enzymatically from ATP that is available at a low cost. Although the He lab has established an efficient enzymatic synthesis method to prepare hundreds of milligrams of c-di-AMP using the diadenylate cyclase DisA from *Bacillus thuringiensis* (Zheng et al., 2013), we propose that DncV may be the most efficient enzyme currently available for the large-scale production of c-di-AMP. DncV can use ATP as its substrate to synthesize c-di-AMP in gram quantities *in vitro* as it is not subject to product inhibition (Launer-Felty and Strobel, 2018; Sun et al., unpublished).

## Data Availability

All datasets generated for this study are included in the manuscript and/or the Supplementary Files.

## Author Contributions

DZ and JZ designed the research. YLv, JZ, and DZ performed most of the experiments, analyzed the data, and wrote the manuscript. All authors contributed to the editing of the manuscript.

## Conflict of Interest Statement

The authors have filed a China patent application (No. 201810545363.1) based on the results reported in this manuscript.

## Footnotes

1 https://www.biolog.de/
