# Supplementary material for: Highly Efficient Preparation of Cyclic Dinucleotides via Engineering of Dinucleotide Cyclases in Escherichia coli
Source: Front Microbiol. 2019 Sep 13;10:2111. doi: 10.3389/fmicb.2019.02111 (PMC6753226; doi:10.3389/fmicb.2019.02111)
Supplement: Supplementary file 1 [file Data_Sheet_1.pdf]

Supplementary Information

Highly efficient preparation of cyclic dinucleotides *via* engineering of dinucleotide cyclases in *Escherichia coli*

Yun Lv[1], Qichao Sun[1], Xiaodan Wang[1], Yi Lu[1], Yao-yao Li[2], Huiqing Yuan[1], Jing Zhu[3*], Deyu Zhu[1*]

[1] Department of Biochemistry and Molecular Biology, School of Basic Medical Sciences, Shandong University, Jinan 250012, China.

[2] Key Laboratory of Chemical Biology (Ministry of Education), School of Pharmaceutical Sciences, Shandong University, Jinan 250012, China.

[3] State Key Laboratory of Microbial Technology, Shandong University, Qingdao 266237, China.

[*] To whom correspondence should be addressed: J.Z.: Ph: +86-532-58631581, Email: zhujing@sdu.edu.cn; D.Z.: Ph:+86-531-88382092-416, Email: zhudeyu@sdu.edu.cn

Supplementary Figures 1-4

Supplementary Table 1-3

Supplementary Methods

Supplementary References

Supplementary Figures

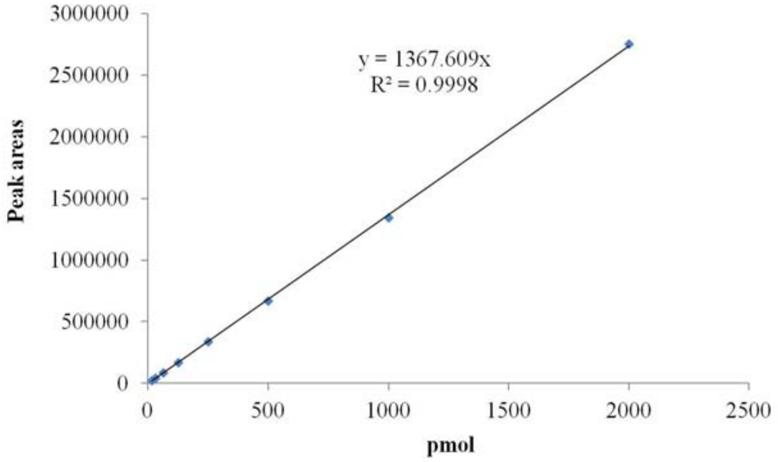

Supplementary Figure 1. Example of a commercial c-di-GMP standard HPLC (UV 254nm) calibration curve. The standard curve was generated by plotting the **peak areas** obtained by the separation of 2 µl of different concentrations of c-di-GMP standards **versus** the corresponding c-di-GMP amounts in **pmol** (for further details see Supplementary Methods). The calibration regression equation was Y=1367.609X (pmol), showing a correlation coefficient (R^2) of 0.9998.

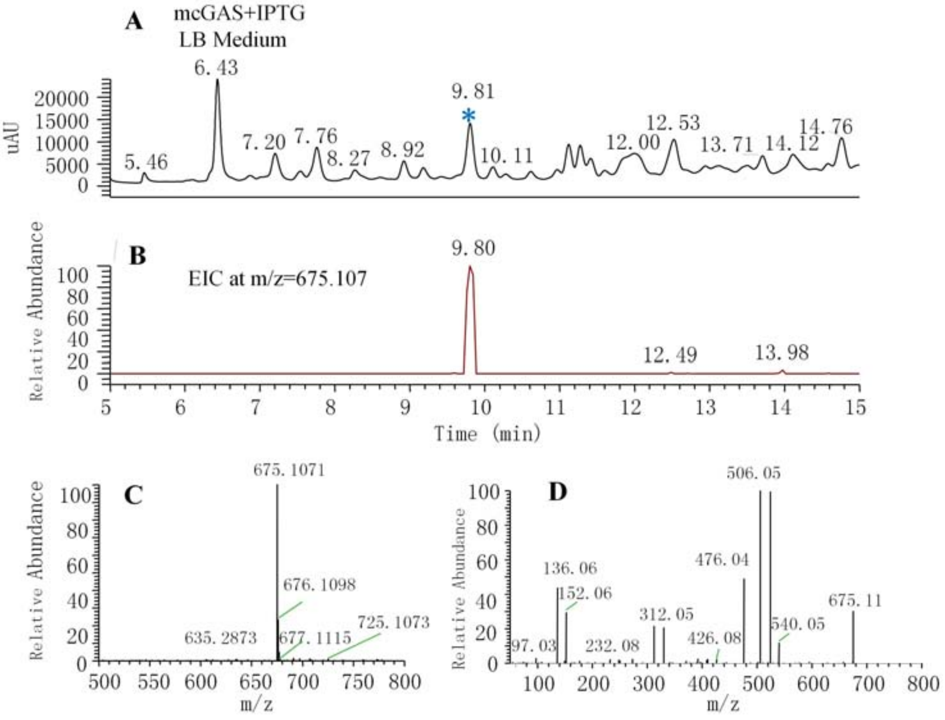

Supplementary Figure 2. Identification of the secreted 2'3'-cGAMP product by HPLC-MS/MS. A. RP-HPLC chromatogram (UV 254 nm) of the cell culture medium (supernatant) after overexpressing mouse cGAS (mcGAS). Blue asterisk indicates a peak

corresponding to the elution of 2’3’-cGAMP. **B.** Extracted ion chromatogram at m/z 675.107 (m/z 675.1070-675.1080) corresponding to the $[M+H]^+$ ion of 2’3’-cGAMP also showed a peak at approximately 9.80 min. **C.** High-resolution mass spectrometric analysis of *peak in chromatogram “**A**”. Peaks at 675.1071 represent the $[M+H]^+$ ion of 2’3’-cGAMP. **D.** Tandem mass spectrum of the 2’3’-cGAMP resulting from higher-energy collision dissociation (HCD) of the precursor ion ($[M+H]^+$ =675.11).

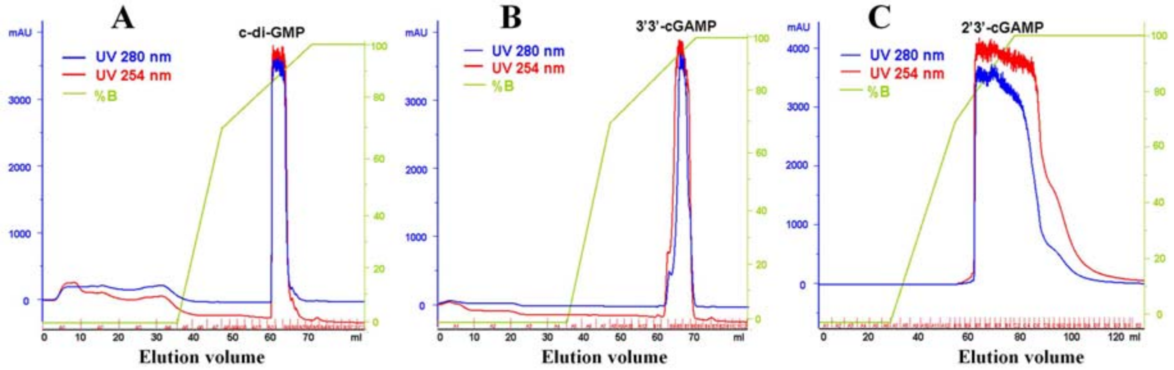

Supplementary Figure 3. Elution curves of c-di-GMP (A), 3’3’-cGAMP (B) and 2’3’-cGAMP (C) from a YMC-Pack C18 ODS-AQ semi-preparative column.

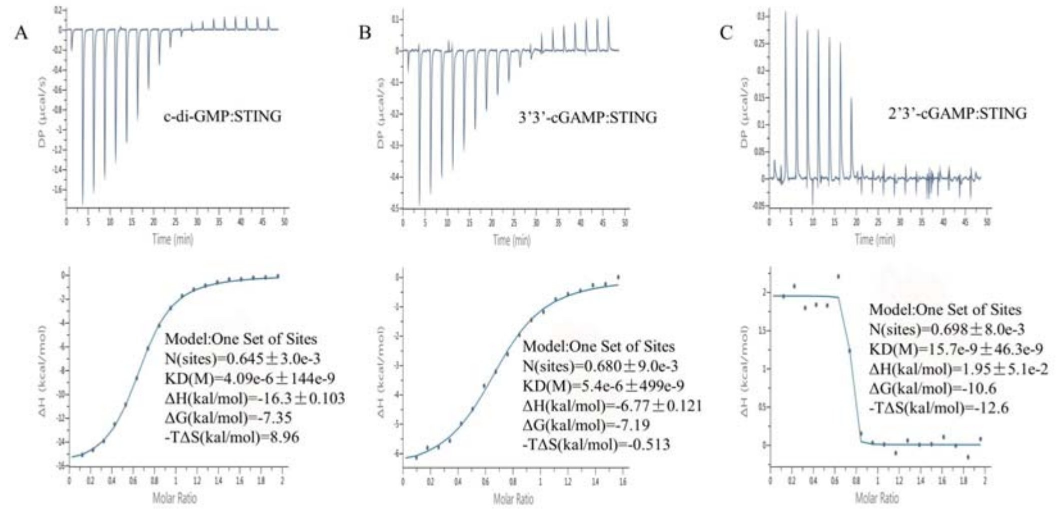

Supplementary Figure 4. CDNs binding by STING as Measured by ITC. The original titration traces and integrated heat data of ITC experiments are shown in the top and bottom panels, respectively. The inserts show the ligand-binding affinities and derived thermodynamic parameters of $STING^{R232/CTD}$ binding to the purified CDNs. The c-di-GMP (A), 3’3’cGAMP (B) or 2’3’cGAMP (C) was titrated into a solution of $STING^{R232/CTD}$.

Supplementary Tables

Supplementary Table 1. The involved genes, vectors and strains in this study.

Purpose	Gene name and the gene encoded protein	vector	Restriction sites	Fusion tag	Host strain
c-di-GMP production	*tDGCm*, Arg158Ala mutant of *T. maritima* DGC (residues 82-248; the wild-type, GenBank accession number NP_229585.1)	pET-15b*	BamHI XhoI	N-terminal 6 X HIS tag	*E. coli* BL21 (DE3)
	Alg44^{PilZ}, PilZ domain of *P. aeruginosa* alginate biosynthesis protein Alg44 (residues 1-122, the wild-type, GenBank accession number AAG06930.1)	pET-28a	NdeI XhoI	N-terminal 6 X HIS tag	*E. coli* BL21 (DE3)
	STING^{CTD}, carboxy terminal domain of human STING^{H232} (residues 149-379, the wild-type, GenBank number NP_938023.1)	pET-30a	NdeI XhoI	C-terminal 6 X HIS tag	*E. coli* BL21 (DE3)
3'3'-cGAMP production	*DncVt*, truncation of *Vibrio cholerae* DncV （residues 1-419, the wild-type, GenBank accession number WP_001901330.1）	pET-22b	NdeI XhoI	C-terminal 6 X HIS tag	*E. coli* BL21 (DE3)
	DncVtm, Thr179Arg mutant of *V. cholera* DncV truncation(residues 1-419)	pET-22b	NdeI XhoI	C-terminal 6 X HIS tag	*E. coli* BL21 (DE3)
	GMK, *Staphylococcus aureus* guanylate kinase (GMK, GenBank accession number WP_000368227.1)	paCYCDu et-1	NdeI XhoI	No	*E. coli* BL21 (DE3)

	NDK, *E. coli* nucleoside diphosphate kinase (NDK, GenBank accession number WP_001696558.1)		BamHI HindIII	N-terminal 6 X HIS tag	*E. coli* BL21 (DE3)
2’3’-cGAMP production	*mcGAS*, mouse Cyclic GMP-AMP synthase (mcGAS, GenBank accession number NP_775562.2)	pET28a-SUMO#	BamHI XhoI	N-terminla 6 X HIS -SUMO	*E. coli* BL21-CodonPlus (DE3)-RIL
STING -immobilized affinity resin	$STING^{LBD}$, ligand binding domain of human STING^{R232} (residues 149-341, the wild-type, GenBank number AVQ94753.1)	pET-30a	NdeI XhoI	C-terminal 6 X HIS tag	*E. coli* BL21 (DE3)
STING binding test	$STING^{R232/CTD}$, carboxy terminal domain of human STING^{R232} (residues 149-379, the wild-type, GenBank number AVQ94753.1)	pET-30a	NdeI XhoI	C-terminal 6 X HIS tag	*E. coli* BL21 (DE3)

*The pET15b vector (Novagen) was modified with a PreScission protease (PPase) site replacing the original thrombin site, and BamHI and XhoI restriction sites replacing the NdeI and BamHI, respectively by PCR mutagenesis techniques.

#The gene encoding *Saccharomyces cerevisiae* SUMO family protein SMT3 (residues 1-97, GenBank accession number NP_010798.1) was subcloned into pET28a vector(Novagen) using NheI and BamHI sites, and thus the pET28a-SUMO vector express a N-terminal 6X HIS-SUMO fusion proteins.

Supplementary Table 2. Primers used in this paper

Prime name	Sequence
**tDGCm F BamHI**	5’-CAGGATCCATGAAAGAGCTGGAG-3’
**tDGCm R XhoI**	5’-AGCTCGAGTCAGCTCAGGCTAAAATA-3’
**STING^{CTD} F NdeI**	5’-CGCCATATGGAAAAAGGGAATTTCAACGTG-3’
**STING^{CTD} R XhoI**	5’-CACTCGAGAGAGAAATCCGTGCGGAG-3’
**STING^{LBD} R XhoI**	5’-GACTCGAGAACCTCTTCCTTTTCCTCCT-3’
**$\mathrm{Alg44}^{PilZ}$ F NdeI**	5’-CACATATGAATACGGCAGTGAACGT-3’
**$\mathrm{Alg44}^{PilZ}$ R XhoI**	5’-CGCTCGAGTCATTCACCTGCCAGGTAGCT-3’
**NDK F BamHI**	5’-AGGATCCGATGGCCATCGAACGTACTTTCA-3’
**NDK R HindIII**	5’-AAAGCTTTCAACGTGTGCGTGGGCACA-3’
**mcGAS F BamHI**	5’-ACGGATCCATGGAAGATCCGCGTAGAAG-3’

**mcGAS R XhoI**	5’-ACGCTCGAGTCAAAGCTTGTCAAAAATTGG-3’
**GMK F NdeI**	5’-CACATATGGACAACGAAAAAGGTCTGCT-3’
**GMK R XhoI**	5’-ACCTCGAGTCATTTTTAGCCTCCAGGAT-3’
**DncVt F NdeI**	5’-CACATATGAGAATGACTTGGAACTT-3’
**DncVt R XhoI**	5’-ACCTCGAGTTCTTGAGCGAAAGCC-3’
**STING(H232R) F**	5’-GCAGACCGGTGACCGTGCTGGCATCAAGG-3’
**STING(H232R) R**	5’-CCTTGATGCCAGCACGGTCACCGGTCTGC-3’
**DncVt(T179R) F**	5’-GGCTGGAAATTTGAAGCTAAGCAGAGATGTGGGAGGATTA-3’
**DncVt(T179R) R**	5’-TAATCCTCCCACATCTCTGCTTAGCTTCAAATTTCCAGCC-3’
**DncVt(T179K) F**	5’-GGAAATTTGAAGCTAAGCAGAAATGTGGGAGGATTAAGATTG-3
**DncVt(T179K) R**	5’-CAATCTTAATCCTCCCACATTTCTGCTTAGCTTCAAATTTCC-3’
**DncVt(T179F) F**	5’-CTGGAAATTTGAAGCTAAGCAGTTTTGTGGGAGGATTAAGATTGAG -3’
**DncVt(T179F) R**	5’-CTCAATCTTAATCCTCCCACAAAACTGCTTAGCTTCAAATTTCCAG -3’
**DncVt(T179H) F**	5’-CTGGAAATTTGAAGCTAAGCAGCATTGTGGGAGGATTAAGATTGAG-3’
**DncVt(T179H) R**	5’-CTCAATCTTAATCCTCCCACAATGCTGCTTAGCTTCAAATTTCCAG-3’
**DncVt(D348S) F**	5’-AGAGAGCCCTGACAGTACCAGCGAAAAGCCACTCTTCCCAC -3’
**DncVt(D348S) R**	5’-GTGGGAAGAGTGGCTTTTCGCTGGTACTGTCAGGGCTCTCT-3’
**DncVt(D348E) F**	5’-GCCCTGACAGTACCGAAGAAAAGCCACTCTTCC-3’
**DncVt(D348E) R**	5’-GGAAGAGTGGCTTTTCTTCGGTACTGTCAGGGC-3’
**DncVt(D348R) F**	5’-AGAGCCCTGACAGTACCCGTGAAAAGCCACTCTTCC-3’
**DncVt(D348R) R**	5’-GGAAGAGTGGCTTTTCACGGGTACTGTCAGGGCTCT-3’

The restriction sites were underlined.

Supplementary Table 3. CDN concentrations detected by HPLC analysis.

	**c-di-GMP**		**3’3’-cGAMP**				**2’3’-cGAMP**
Cells expressing	tDGCm	tDGCm and $STING^{CTD}$	DncVt	DncVt and NDK and GMK	DncVtm	DncVtm and NDK and GMK	Sumo-mcGAS
1L Medium	LB					TB	Modified M9
Induction temperature and time	37 ˚C, 20h		18 ˚C, 20h				37 ˚C, 20h
Wet weight of cell pellets (mg)	5,4 35	5,018	5,012	4,964	4,885	6,901	N/A
Resuspension volume	180 ml						1 L (culture supernatant)
Injection volume for HPLC	2 µl						
Chromatogram related to Supplementary Figure 1	A Upper	A Bottom	B Upper first	B Upper second	B Upper third	B Bottom	C Bottom
Peak areas (uAU×s)	2,943,275	2,518,310	526,185	511,478	485,275	1,528,646	627,405

CDN concentrations (nmol mg^{-1})	35.64*	33.03*	6.54&	6.43&	6.15&	13.81&	217.04 μM#

Based on the linear regression equation obtained from the HPLC standard curve of c-di-GMP (Supplementary Figure 1) and the different extinction coefficients between c-di-GMP and cGAMPs, each CDN concentration was calculated using the following different equation.

* the equation for c-di-GMP concentration:

c-di-GMP (nmol mg^{-1})= [(Peak area) X 180 (ml)] /[1367.609 X 2 (μl) X (cell wet weight) (mg)];

& the rough equation for 3'3'-cGAMP concentration:

3'3'-cGAMP (nmol mg^{-1})= [(Peak area) X 180 (ml) X 23700] /[1367.609 X 2 (μl) X (cell wet weight) (mg) X 25050];

\# the rough equation for 2'3'-cGAMP concentration:

2'3'-cGAMP(μM)= [(Peak areas) X 23700]/[1367.609 X 2 (μl) X 25050].

where peak areas (unit, uAU×s) was determined using the software LCsolution 1.26 SP 1 of the HPLC system, and 180, 2, 23700 and 25050 represent the resuspension volume (ml) for cell pellets from 1L culture, the injection volume (μl) for HPLC analysis, the molar extinction coefficient ε for c-di-GMP and two cGAMPs, respectively.

Supplementary Methods

Preparation of CDNs.

1. Production of CDNs:

(1) c-di-GMP: The expression plasmids pET15b carrying the *tDGCm* gene and pET30a carrying the *STING*^{CTD} gene (Supplementary Table 1) were co-transformed into *E. coli* BL21 (DE3) strain. Overnight cultures were diluted 100-fold into fresh LB medium with 100 ug/ml ampicillin and 50 ug/ml kananmycin and then grown at 37 °C to an OD600 of 0.8-0.9. The cultures were supplemented with 0.1 mM IPTG and then grown for an additional 20 hours at 37 °C (200 rpm). Cells were pelleted by centrifugation at 4500 rpm for 30 minutes at 4 °C, and the cell pellets were retained and stored at -80 °C for lysis.

(2) 3'3'-cGAMP: The expression plasmids pET22b carrying the *DncVtm* gene and paCYCduet-1 carrying the *NDK* and *GMK* gene (Supplementary Table 1) were co-transformed into *E. coli* strain BL21 (DE3). Overnight cultures in LB medium were diluted 100-fold into fresh TB medium with 100 ug/ml ampicillin and 25 ug/ml chloramphenicol, and grown at 37 °C to an OD600 of 1.1- 1.2 before being induced with 0.1 mM IPTG at 18 °C, 200 rpm for 20 hours. Cells were pelleted by centrifugation at 4500 rpm for 30 minutes at 4 °C, and the cell pellets were retained and stored at -80 °C for lysis.

(3) 2'3'-cGAMP: The expression plasmids pET28a-SUMO carrying the *mcGAS* gene (Supplementary Table 1) was transformed into *E. coli* strain BL21-CodonPlus (DE3)-RIL. As described above, the cells from 1 volume of the overnight culture was pelleted by centrifugation, washed twice and resuspended in 1 volume of M9 minimal medium, and then transferred to 250 volume of the fresh M9 minimal salts supplemented with 50 ug/ml kananmycin, 0.8% glucose, 5 mM $MgSO_4$, 0.1 mM $CaCl_2$ and 0.01 mM fresh ferrous sulphate. The culture was grown at 37 °C to an OD600 of 0.8- 0.9 (about 10 h), and then supplemented with 0.1 mM IPTG followed by incubation at 37 °C, 200 rpm for 20 hours. Then the bacterial cultures were centrifuged at 4500 rpm for 30 minutes at 4 °C, and the culture supernatants were retained and stored at -80 °C for later use.

2. Purification of CDNs:

(1) c-di-GMP or 3'3'-cGAMP: the harvested cell pellets harboring c-di-GMP or 3'3'-cGAMP from 0.5 L culture were thoroughly resuspended in 90 ml of resuspension buffer [20 mM Tris-HCl (pH8.0), 200 mM NaCl] supplemented with 0.2 mg/ml DNase and 0.1 mM PMSF, and then lysed by using a mini ultra high pressure cell disrupter (JNBIO, China) with a pressure midpoint of ~ 1200 Bar, at 4 °C. Cell debris was removed by centrifugation (12000g and 4 °C for 50 min), the supernatant was retained, heated at 100 °C for 15 min, centrifuged again at 12000g and 4 °C for 30 min, filtered through a PES sterile syringe filter (pore size 0.22 μm, Millipore, USA), and then diluted by adding 2 volumes of the resuspension buffer. Then ~270 ml of sample supernatant was loaded onto a column including 50 ml of the $STING^{LBD}$ immobilized affinity resin prepared by us (this is described as above) at 10 °C and gravitationally passed through. The flow-through was retained for reloading in the next cycle. After sample loading, the $STING^{LBD}$ immobilized affinity column was washed with 500 ml of 300 mM sodium acetate, and the washed-out solution was also retained for loading in the last cycle. The target c-di-GMP or 3'3'-cGAMP was eluted from the $STING^{LBD}$ immobilized affinity column with 0.1 M urea until the elution showed very little of CDN with less than 5 ng/μL of nucleic acid concentration, which was determined by a k5600 spectrophotometer (Beijing Kaiao Technology Development Company, Beijing, China). Usually, the elution volume was about 200 ml with 100-160 ng/μL of nucleic acid concentration. After the first elution, the $STING^{LBD}$ immobilized affinity column was re-preconditioned for reuse with 0.5 L of 2 M urea and 0.5 L of the resuspension buffer. Both the flow-through and washed-out

solution of the first cycle were reloaded onto the STING^{LBD} immobilized affinity column, which was then washed with 500 ml of 300 mM sodium acetate and eluted with 200 ml of 0.1 M urea again. To ensure that almost all target CDN was captured by the STING^{LBD} immobilized affinity column, the flow-through and washed-out solution of the second cycle were processed one more time using the same procedure as the second cycle. Then all three elutions (~600 ml) were combined together, acidified to about pH3.0 with ~12 ml of acetic acid (final concentration 2% (v/v)), filtered through cellulose filters (pore size 0.45 μm), and then incubated with 50 ml of preconditioned macroporous adsorption resin (Diaion SP207, Mitsubishi Chemical Industries Ltd., Japan), which was pretreated by firstly soaking in 100 ml of ethanol for 24h, and then consecutively washing with 250 ml of ethanol, 250 ml of water, 250 ml of 0.5 M hydrochloric acid, extensive water (to neutral pH), 250 ml of 0.5 M sodium hydroxide, extensive water (to neutral pH), and finally pre-acidifying to about pH3.0 with 1% (v/v) acetic acid (Chang et al., 2012). After incubating with the sample for 1h, with interval stirring, the resin SP207 was transferred to a 30-cm-long column with an inner diameter of 2.2 cm, and then washed with 350 ml of 1% (v/v) acetic acid and 50 ml of water. Elution of the target CDN was performed by using 500 ml of 10% (v/v) ethanol. To increase the yield, the flow-through and washed-out solution from the first round of SP207 extraction were mixed (~ 1L), and processed one more time using the same procedure as the first round with another 50 ml of preconditioned SP207 resin. Then two elutions (~1 L) of SP207 resin were combined together, dried by rotary evaporation, and then resuspended in 35 ml water (pH 9, adjusted with ammonium hydroxide), filtered through a PES sterile syringe filter (pore size 0.22 μm), and acidified to pH 2.1 with HCl (this step is important for ensuring both complete dissolution of CDN and follow-up step), and loaded onto a YMC-Pack C18 ODS-AQ semi-preparative column (10 x 250 mm, 5 μm, YMC) pre-equilibrated in AQ-A buffer (5 mM ammonium formate pH 2.1, adjusted with formic acid) on a fast protein liquid chromatography system (FPLC) (Äkta Explorer, GE Healthcare) equipped with a UV-900 detector (A254 and A280) at a flow rate of 0.5 ml/min. After loading the sample, the column was washed with more than 36 ml of AQ-A buffer, and then eluted using two-step gradient elution (12 ml from 0% B to 70% B and 24 ml from 70% B to 100% B, AQ-B buffer, 10% acetonitrile) (Zhu et al., 2014). The main peak fractions corresponding to the target CDNs were pooled, neutralized by ammonium hydroxide, filtered through a PES sterile syringe filter (pore size 0.22 μm), dried by rotary

evaporation, resuspended in 4 ml of 0.5 ‰(v/v) ammonium hydroxide solution, and freeze-dried by a vacuum-freezing dryer (Heto-Holten, Denmark). Finally, from 0.5 L culture, we obtained 36.53 ± 2.18 and 15.89 ± 1.89 milligrams of the diammonium salts (white powder) of c-di-GMP and 3'3'-cGAMP, which correspond to overall yields of > 56%, > 38% of the initial production in the harvested cells, respectively.

(2) 2'3'-cGAMP: the culture supernatant harboring 2'3'-cGAMP from 0.5 L culture was filtered through cellulose filters (pore size 0.45 μm) to produce the sample supernatant, which then was purified in the same way (sequential chromatography) as the c-di-GMP and 3'3'-cGAMP described above, except that there are three differences: **1)** the elution solution of the $STING^{LBD}$ immobilized affinity column was 1.5 M urea; **2)** the loading sample of the YMC-Pack C18 ODS-AQ semi-preparative column was acidified to pH 4.5 with HCl before loading, and the corresponding AQ-A buffer of the C18 ODS-AQ semi-preparative column was 5 mM ammonium formate pH 4.5 (adjusted with formic acid); **3)** the two-step gradient elution of the YMC-Pack C18 ODS-AQ semi-preparative column were 26.4 ml from 0% B to 70% B and 24 ml from 70% B to 100% B. Finally, from 0.5 L culture, we obtained 42.80 ± 1.67 milligrams of the diammonium salts (white powder) of 2'3'-cGAMP, which correspond to overall yields of > 53% of the initial production in the culture supernatant.

Supplementary References

Chang X. L., Wang D., Chen B. Y., Feng Y. M., Wen S. H., Zhan P. Y. (2012). Adsorption and desorption properties of macroporous resins for anthocyanins from the calyx extract of roselle (Hibiscus sabdariffa L.). *J Agric Food Chem*, 60(9):2368-2376. doi:10.1021/jf205311v

Zhu D., Wang L., Shang G., Liu X., Zhu J., Lu D., et al. (2014). Structural biochemistry of a Vibrio cholerae dinucleotide cyclase reveals cyclase activity regulation by folates. *Mol Cell*, 55(6):931-937. doi:10.1016/j.molcel.2014.08.001
